# Cell Wall Invertase 4 Governs Sucrose–Hexose Homeostasis in the Apoplast to Regulate Wood Development in Poplar

**DOI:** 10.3390/plants14091388

**Published:** 2025-05-04

**Authors:** Jing Lu, Qiao Ren, Qilin Wang, Yaqi Wen, Yanhong Wang, Ruiqi Liang, Dingxin Ran, Yifeng Jia, Xinyu Zhuo, Jiangtao Luo, Xianqiang Wang, Keming Luo

**Affiliations:** 1 Chongqing Key Laboratory of Innovative and Utilization of Forest Germplasm Resources, Integrative Science Center of Germplasm Creation in Western China (Chongqing) Science City, School of Life Sciences, Southwest University, Chongqing 400715, China; jinglu2021@swu.edu.cn (J.L.);; 2 Key Laboratory of Eco-Environments of Three Gorges Reservoir Region, Ministry of Education, School of Life Sciences, Southwest University, Chongqing 400715, China

**Keywords:** poplar, xylem development, SCW, sugar metabolism, sugar signaling

## Abstract

In perennial trees, wood development is a carbon-demanding process, pivotal for secondary cell wall (SCW) formation and xylem development. Sugars, functioning both as carbon substrates and signaling molecules, orchestrate cambial proliferation and xylem differentiation. However, few molecular candidates involved in the sugar-mediated regulation of wood development have been characterized. Cell wall invertases (CWINs), a subclass of the invertase enzyme family localized in the apoplastic space, catalyze the irreversible hydrolysis of sucrose into glucose and fructose, thereby governing carbon allocation in sink tissues. Here, *PtoCWIN4* shows preferential expression in the stem of *Populus tomentosa* and has a high efficiency in sucrose cleavage activity. We demonstrated that the knockout of *PtoCWIN4* results in stunted growth, aberrant branching patterns, and compromised secondary xylem formation. In contrast, mutant lines displayed enhanced SCW thickness accompanied by elevated cellulose and hemicellulose accumulation. Following this, the knockout of *PtoCWIN4* led to impaired carbon partitioning from sucrose to hexose metabolites during wood development, corroborating the enzyme’s role in sustaining sucrose hydrolysis. Collectively, these findings establish *PtoCWIN4* as a master regulator of sucrose-to-hexose conversion, a metabolic gateway critical for balancing structural biomass production and developmental growth during wood formation.

## 1. Introduction

Forests play a critical role in mitigating climate change by absorbing substantial amounts of CO_2_ emissions, with a significant proportion of sequestered carbon stored in wood. Wood formation involves the division and differentiation of cambium cells into xylem cells, followed by cell expansion and secondary cell wall (SCW) deposition. This process requires substantial carbon resources, primarily derived from sugars produced during photosynthesis. Sucrose, the main form of carbon transport in plants, is translocated via the phloem to the cambium and xylem, providing energy and carbon skeletons for cell division, expansion, and SCW biosynthesis. Beyond serving as a primary energy source and structural component, sugars also act as signaling molecules regulating plant growth and development [1,2,3,4]. Sucrose, as both a carbon source and a signaling molecule, modulates cambium activity and xylem differentiation [5,6,7,8]. However, the molecular mechanisms by which sugars regulate wood development remain poorly understood.

Previous studies have reported that sucrose transporter 3 (SUT3) in hybrid aspen (*Populus tremula* × *tremuloide*) is expressed during SCW formation in developing wood [9]. RNA interference (RNAi)-mediated silencing of *SUT3* in the SCW-forming region of developing wood reduced carbon incorporation into cell walls, indicating that SUT3 facilitates apoplastic sucrose uptake [9]. These findings collectively establish that sucrose must be actively imported into developing fiber cells via SUT3-mediated transport to fuel SCW biosynthesis. Critically, this mechanistic framework highlights that the apoplastic sucrose pool in the cell wall space serves as a rate-limiting determinant of cellulose and hemicellulose deposition efficiency during wood formation. Similarly, the sucrose efflux carrier SWEET7, expressed on the plasma membrane of phloem cells, enhances carbon allocation to the xylem when overexpressed [10]. The vacuolar transporter PtaSUT4 has also been shown to influence carbon partitioning between source leaves and xylem sinks in *Populus tremula* × *alba* [11]. Additionally, Gerber et al. (2014) [12] demonstrated that RNAi-mediated suppression of sucrose synthase (SUS) activity (to 4% of wild-type levels) in hybrid poplar (*P. tremula* × *tremuloide*) reduced carbon incorporation into cellulose, hemicellulose, and lignin, leading to decreased wood density. However, no specific inhibition of cellulose biosynthesis was observed. In developing wood of hybrid poplar (*P. tremula* × *tremuloide*), reducing cytosolic invertase (CIN) activity by 38–55% via SCW promoter-driven RNAi constructs resulted in a 9–13% decline in crystalline cellulose levels, suggesting the involvement of invertase pathways in cellulose biosynthesis [13]. While these findings underscore the pivotal role of sugar allocation and metabolism in wood formation, the discovery of novel regulatory components governing these processes remains a critical priority.

Studies have revealed that long-distance-transported sucrose unloaded into developing xylem undergoes sugar metabolism to provide substrates for secondary cell wall (SCW) biosynthesis. The SCW, serving as the terminal sink tissue in wood, primarily consists of polysaccharides such as cellulose and hemicellulose [14,15]. The production of UDP-glucose, a critical substrate for SCW synthesis, occurs via two pathways: reversible hydrolysis by sucrose synthases (SUSs) that catalyze the conversion of sucrose to fructose and UDP-glucose [16]; irreversible hydrolysis by invertases (INVs) that cleave sucrose into fructose and glucose [17]. Research demonstrates that disrupting sucrose hydrolysis alters UDP-glucose levels and impairs SCW formation. For instance, suppressing SUSs’ activity in *Arabidopsis thaliana*, *P. tremula* × *tremuloide*, and other species reduces SCW deposition [13,18,19]. The RNAi-mediated knockdown of INVs in *P. tremula* × *tremuloide* decreases crystalline cellulose content [13]. In cotton, SUSs localized to the plasma membrane may directly interact with cellulose synthase complexes (CESAs), providing UDP-glucose for cellulose synthesis via sucrose hydrolysis [20,21,22]. Similarly, impaired sucrose supply in the *Arabidopsis* starch-deficient mutant (*pgm*) disrupts cellulose biosynthesis [23]. These findings highlight the critical role of sucrose homeostasis in SCW synthesis. Recent studies further show that perturbing intracellular sugar balance in *Arabidopsis* xylem cells suppresses SCW synthesis-related gene expression while upregulating key SCW regulators (e.g., *SND1*, *MYB46*/*83*, and *VNDs*) [24]. This suggests that sugars not only act as substrates but also modulate the expression of SCW regulatory factors. However, the molecular mechanisms underlying the sugar-mediated regulation of these factors remain unclear.

INVs—known as sucrases or β-D-fructofuranosidases—irreversibly hydrolyze sucrose into glucose and fructose, playing a pivotal role in carbon utilization [13]. INVs are categorized into three distinct groups based on their subcellular localization and enzymatic properties: cell wall invertases (CWINs); vacuolar invertases (VINs); and cytoplasmic invertases (CINs) [25,26]. Classification by pH optimization reveals that CWINs and VINs function as acidic invertases (optimal pH: 3.5–5.5), whereas CINs operate within neutral/alkaline pH ranges (6.5–8.0), reflecting their distinct biochemical roles in cellular compartments [27]. In poplar, 16 neutral invertases (CINs) and 8 acidic invertase genes have been identified, including 5 encoding CWINs and 3 encoding soluble vacuolar enzymes [28]. CWIN-mediated sucrose hydrolysis in the apoplast is essential for unloading sucrose from phloem sieve elements into the apoplast [29]. By hydrolyzing apoplastic sucrose, CWINs establish sink strength through increased osmotic pressure in sink tissues, facilitating sucrose transport from source leaves to sinks such as wood [30,31]. In wood development, cellulose deposition correlates with sucrose unloading efficiency, implicating CWINs in regulating carbon allocation to the xylem. Overexpression of the wheat *TaCWI-B1* gene thickened cell walls and elevated pectin and cellulose content while enhancing pest resistance via interaction with α-galactosidase (TaGAL) [32]. However, the role of CWINs in structural carbohydrate synthesis, particularly in woody plants, remains underexplored.

Beyond metabolic functions, CWINs participate in sugar signaling [1]. For instance, CWIN overexpression in *Arabidopsis* shoot apical meristems accelerated flowering and increased inflorescence branching [2]. Elevated CWIN activity in tomato suppresses programmed cell death under heat stress [33]. The miniature seed phenotype of maize CWIN-deficient mutants could not be rescued by exogenous hexose supplementation [34]. Notably, developmental defects in pollen caused by invertase mutations were not rescued by exogenous sugar [27], and CWIN suppression-induced ovule abortion did not activate carbon starvation genes or recover with sugar supplementation. These findings suggest that CWINs possess signaling roles independent of their metabolic functions. Nevertheless, the mechanisms underlying the CWIN-mediated regulation of vascular development in woody plants remain unclear.

In this study, we investigate the role of *PtoCWIN4* in wood formation of *P. tomentosa* through an integrated approach employing CRISPR-Cas9-mediated knockout, wood anatomical analysis, expression analysis, soluble sugar quantifications, and enzyme activity measurements. We aim to determine whether *PtoCWIN4* hydrolytic activity contributes to carbon supply for cellulose synthesis or participates in signaling pathways regulating vascular tissue development in wood. This work provides the first functional dissection of CWIN in wood development, offering novel insights for the genetic improvement of forest trees.

## 2. Results

### 2.1. Characterization of PtoCWIN4 from P. tomentosa

**Figure 1:**
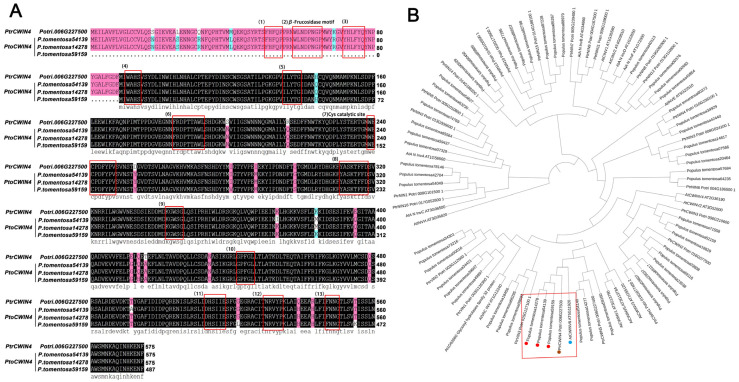
Sequence alignment and phylogenetic analysis of amino acid sequences of *CWIN4* from *A. thaliana*, *P. trichocarpa*, and *P. tomentosa*. (**A**) Sequence alignments of *PtoCWIN4* with *PtrCWIN4*. Amino acid sequences were aligned with the software DNAMAN 8. The boxed region indicates the 13 well-conserved regions from known acid invertases. (**B**) Phylogenetic tree of invertase proteins from *A. thaliana*, *P. trichocarpa*, and *P. tomentosa*. In the rectangular box, the *CWIN4* genes from three species are shown: *A. thaliana* (blue circle), *P. trichocarpa* (brown circle), and *P. tomentosa* (red circle).

To understand the phylogenetic relationships among the invertases in *Arabidopsis*, *Populus trichocarpa*, and *P. tomentosa*, an ML phylogenetic analysis was performed based on the full-length sequences of amino acids from the three species using PhyML 3.0.—five CWIN genes of *P. trichocarpa* were identified, which is identical to a previous study by Bocock et al. (2008) [28]. Each gene in *P. trichocarpa* has three homologous sequences existing in *P. tomentosa* (Figure 1B). 

**Figure 2:**
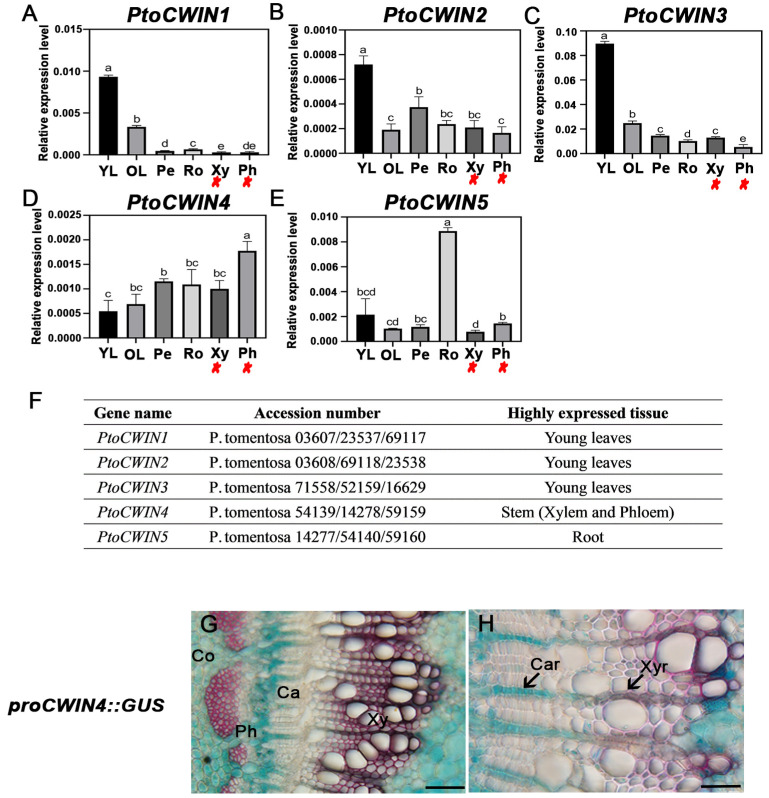
Expression patterns of *PtoCWIN4* in poplar. (**A**–**E**) RT-qPCR analysis of tissue-specific expression profiles of *PtoCWIN* genes. (**F**) High-expression tissues for each *PtoCWIN* gene. Different letters denote statistical differences (*p* < 0.05) as determined by one-way ANOVA. *n* = 3 for technical replicates. (**G**,**H**) Histological staining of GUS reporter driven by the *PtoCWIN4* promoter in poplar stems. The 7th internodes of 2-month-old poplar plants were cross-sectioned for GUS staining. Arrows indicate ray cells with GUS staining. Data shown represent three biological replicates (three technical replicates per replicate). YL, young leaves; OL, old leaves; Pe, petiole; Ro, root; Ph, phloem; Xy, xylem; Co, cortex; Ca, cambium; Phr, phloem ray; Car, cambium ray; Xyr, xylem ray. Red asterisks indicate gene expression in stem (xylem and phloem). Scale bars = 100 μm (**F**), 50 μm (**G**).

Utilizing scRNA-seq data from Sundell et al. (2017) [35], *PtrCWIN4* has been implicated as being highly expressed in the stem, and sequence alignment revealed that *PtoCWIN4* shared high identity with *PtrCWIN4* and 13 well-conserved regions from known acid invertases were identified in the sequences (Figure 1A). The RT-qPCR was employed to validate its stem-elevated expression compared to the other *PtoCWIN* genes (Figure 2A–F), suggesting a potential functional role in wood development. Further, to investigate the expression pattern of *PtoCWIN4*, we isolated the *PtoCWIN4* promoter to drive the GUS reporter gene, generating *ProPtoCWIN4:GUS* transgenic lines. GUS staining of *ProPtoCWIN4:GUS* transgenic plants revealed high expression in the stems; *PtoCWIN4* in the stem’s seventh internode through histological examination shows significant expression in the cortex, the centripetal part of phloem cells, phloem ray cells, ray initials in cambium, xylem ray cells, and the pit (Figure 2G,H). Notably, *PtoCWIN4* transcript levels were particularly elevated at the phloem–ray connection site, which acts as the sucrose phloem unloading site, suggesting a potential role for *PtoCWIN4* in regulating sucrose metabolism in wood.

### 2.2. Construction and Identification of Transgenic Poplars

**Figure 3:**
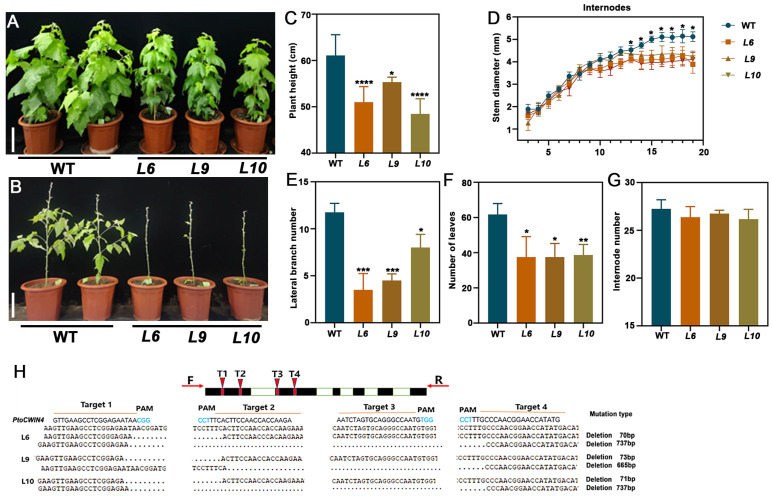
Knockout of *PtoCWIN4* alters the overall development of *P. tomentosa.* (**A**,**C**) Dwarf phenotypes of 2-month-old *PtoCWIN4* mutant lines. (**B**,**E**,**F**) Reduced branches (**B**,**E**) and leaves (**F**) in *PtoCWIN4* mutant lines. (**D**) Decreased stem diameter and internode number (**G**) in *PtoCWIN4* mutant lines. (**H**) Diagram of CRISPR/Cas9 target sites of *PtoCWIN4* and determination of the mutations in the coding region of *PtoCWIN4* generated by the CRISPR/Cas9 system. This panel illustrates the mutations introduced into the *PtoCWIN4* coding region by the CRISPR/Cas9 system. The text on the right summarizes the mutation details from three independent CRISPR/Cas9-generated lines (L6, L9, and L10). T refers to target. Data represent three independent biological replicates, with error bars showing SD. Statistical differences were determined by two-tailed Student’s *t*-test (* *p* < 0.05; ** *p* < 0.01; *** *p* < 0.001; **** *p* < 0.0001, *n* = 3). Scale bars: 20 cm (**A**,**B**).

To elucidate the role of *PtoCWIN4* in poplar, we successfully generated *PtoCWIN4* knockout mutants (#L6, #L9, and #L10) in *P. tomentosa* using a CRISPR/Cas9-based genome-editing approach. The target gene *PtoCWIN4* (*P. tomentosa* 54139/14278/59159) was disrupted through precise engineering of four sgRNA target sites (Figure 3H). Four 20-base pair guide sequences, each followed by a protospacer adjacent motif (PAM: 5′-NGG-3′), were strategically designed within the first two exons of *PtoCWIN4*. The knockout construct (*PtoCWIN4*-KO) was introduced into *P. tomentosa* via *Agrobacterium*-mediated leaf disc transformation. Through rigorous screening, three independent homozygous mutant lines (#L6, #L9, and #L10) were established. Sequencing analysis revealed distinct deletion patterns at all four sgRNA-targeted loci across the mutant lines: In line L6, two distinct editing outcomes were observed—(i) a 70-base pair deletion spanning target sites T1 and T2; (ii) a large 737-base pair deletion encompassing all four target sites, confirming homozygous mutations. Lines L9 and L10 exhibited similar deletion patterns (Figure 3H). These mutations induced frameshifts or premature stop codons in *PtoCWIN4*, validating the functional knockout of *PtoCWIN4* via CRISPR/Cas9.

### 2.3. Knockout of PtoCWIN4 Alters the Phenotype of Poplar

To delineate the biological function of PtoCWIN4 in poplar, we performed phenotypic comparisons between *PtoCWIN4* mutants and wild-type plants (WT). The *PtoCWIN4* mutant plants exhibited significantly reduced growth (Figure 3A). Knocking out *PtoCWIN4* resulted in a 9.3–20.7% reduction in plant height and a 14.8–22.2% reduction in stem diameter (Figure 3C,D), along with a 31.9–70.2% reduction in the lateral branch number (Figure 3B,E) and 37.4–39.3% reduction in the number of leaves. However, the number of internodes showed no significant changes due to *PtoCWIN4* knockout (Figure 3G).

### 2.4. PtoCWIN4 Plays a Functional Role in Regulating Xylem Development in Poplar

**Figure 4:**
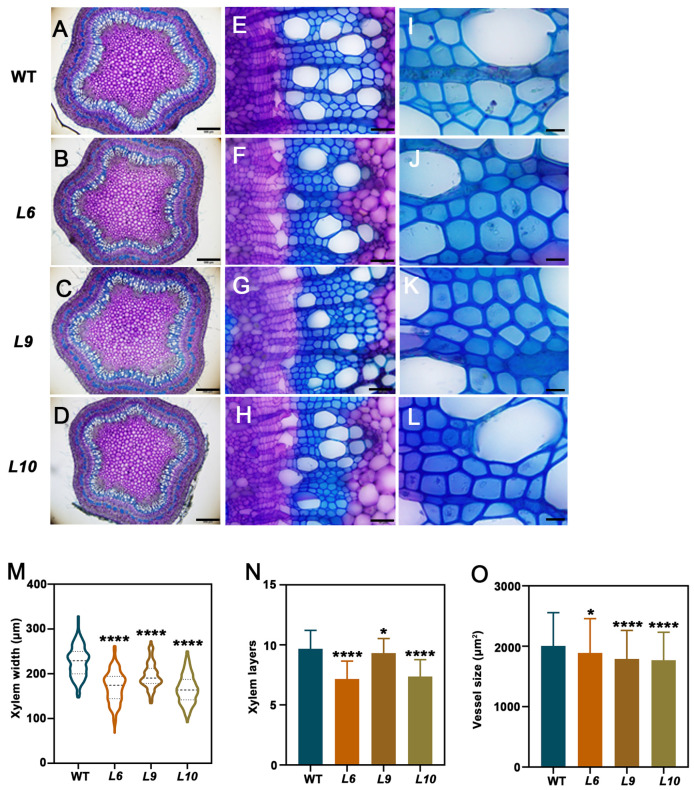
Wood phenotypes resulting from knockout *PtoCWIN4* in *P. tomentosa*. (**A**–**L**) Cross-sections of the 7th internode stained with toluidine blue, highlighting xylem (Xy). Xylem width measurements (**M**), quantification of secondary xylem cell layers (**N**), and vessel size measurements (**O**) in WT and *PtoCWIN4* mutants using ImageJ (https://imagej.net/ij/) on toluidine blue-stained sections. Data represent three independent biological replicates, with error bars showing SD. Statistical differences were determined by two-tailed Student’s *t*-test (* *p* < 0.05; **** *p* < 0.0001, *n* = 3). Scale bars = 500 μm (**A**–**D**); 50 μm (**E**–**H**); 15 μm (**I**–**L**).

To investigate the role of *PtoCWIN4* in wood formation, toluidine blue O staining was performed. As shown in Figure 4A–L, secondary xylem development was significantly suppressed in the *PtoCWIN4* mutant, evidenced by a 14–27.1% reduction in the xylem width and a 3.7–26% decrease in the number of xylem cell layers compared to the WT (Figure 4M,N). Additionally, the average vessel lumen diameter decreased by 6.1–11.8% (Figure 4O). These results collectively indicate that *PtoCWIN4* knockout attenuates wood formation in poplar.

### 2.5. PtoCWIN4 Knockout Alters the Composition of Secondary Cell Walls in Xylem

**Figure 5:**
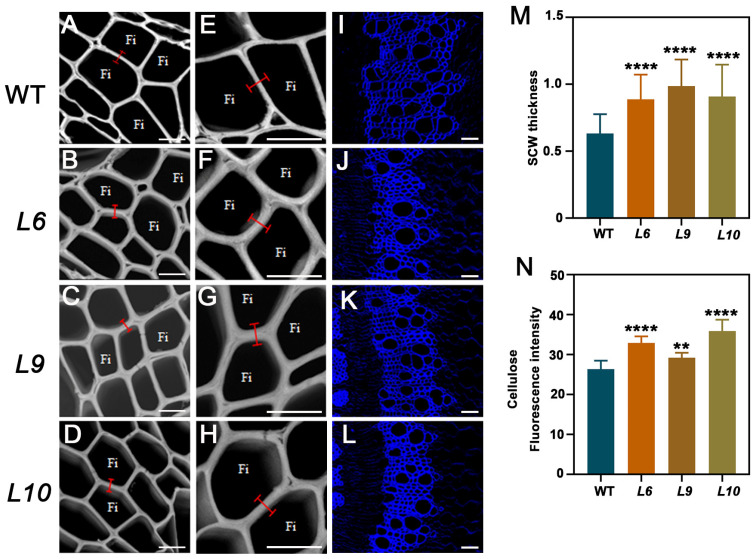
Reduced xylem secondary cell wall formation in *PtoCWIN4* mutant plants. (**A**–**H**) SEM pictures of secondary cell wall thickness of *PtoCWIN4* mutant and WT. (**I**–**L**) Calcofluor white staining of cellulose in SCW. (**M**) SCW thickness measurements in WT and *PtoCWIN4* mutants using ImageJ on SEM pictures. (**N**) Fluorescent intensity quantification in WT and *PtoCWIN4* mutants using ImageJ on calcofluor-white-stained sections. Data represent three independent biological replicates, with error bars showing SD. Statistical significance was evaluated using a two-tailed Student’s *t*-test (** *p* < 0.01; **** *p* < 0.0001). Statistical group sizes: *n* > 30 for SCW thickness measurements, *n* > 10 for fluorescent intensity quantification. Scale bars = 10 μm (**A**–**D**); 10 μm (**E**–**H**); 50 μm (**I**–**L**).

**Figure 6:**
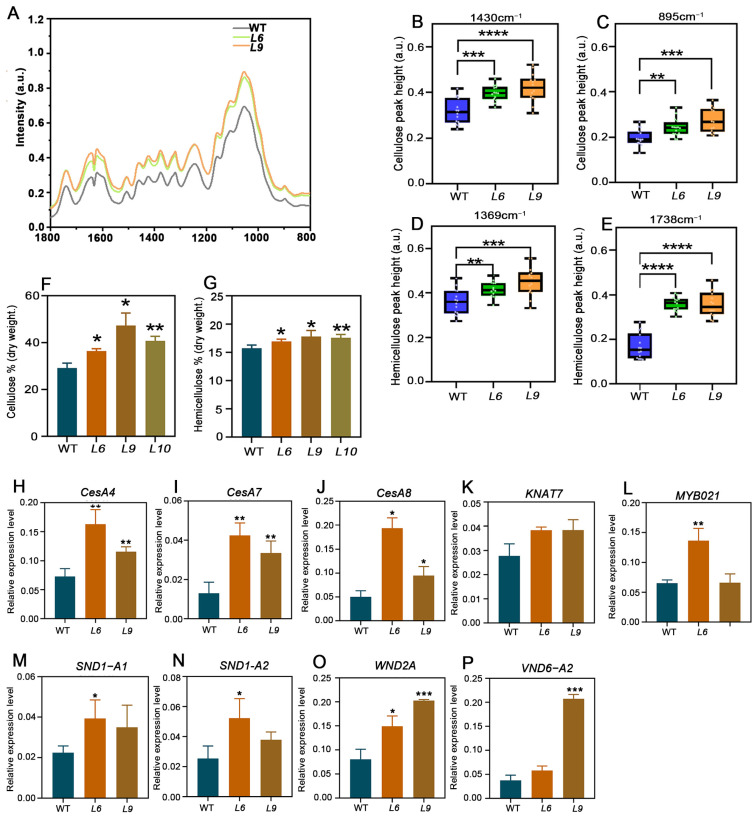
Analysis of xylem secondary cell wall components in *PtoCWIN4* mutant plants. (**A**) FTIR spectra of xylem tissue from the 7th internode of 2-month-old poplar grown in soil. Spectra were baseline-corrected and area-normalized within 1800–800 cm^−1^. Average spectra for WT and *PtoCWIN4* mutants were obtained from three independent biological replicates. (**B**–**E**) Boxplots displaying absorbance values for cellulose (1430 cm^−1^, 895 cm^−1^) and hemicellulose (1738 cm^−1^, 1369 cm^−1^). (**F**,**G**) Quantitative measurements of cellulose (**F**) and hemicellulose (**G**). (**H**–**P**) Relative expression levels of cellulose synthesis genes *CesA4*/*7*/*8* in WT and *PtoCWIN4* mutants determined by RT-qPCR. (K–P) Relative expression levels of master regulators for SCW synthesis *KNA7*, *MYB021*, *SND1-A1*, *SND1-A2*, *WND2A*, *VND6-A2*. Data represent three independent biological replicates, with error bars showing SD. Statistical significance was evaluated using a two-tailed Student’s *t*-test (* *p* < 0.05; ** *p* < 0.01; *** *p* < 0.001; **** *p* < 0.0001). Boxplot statistical group sizes: *n* > 30 for FTIR, *n* = 3 for other experiments.

To our surprise, toluidine blue O staining revealed a significant increase in xylem cell wall thickness. To determine the impact of *PtoCWIN4* on secondary cell wall formation, SEM (scanning electron microscopy) analyses were conducted to observe the thickness of SCW, and knocking out *PtoCWIN4* resulted in a 40.4–56.2% increase in SCW thickness (Figure 5A–H,M). Furthermore, Fourier-transform infrared spectroscopy (FTIR) was employed to analyze the biochemical composition of the xylem secondary cell walls. The spectral profiles for the *PtoCWIN4* mutant and the WT exhibited differences at 895^−1^, 1369^−1^, 1430^−1^, and 1738^−1^ in fingerprint regions corresponding to cellulose and hemicelluloses (Figure 6A–E). The absorbance associated with cellulose and hemicelluloses showed significant differences at 895 cm^−1^ and 1430 cm^−1^, respectively, which are associated with the amorphous region [36] and crystalline cellulose, respectively [37]. The wavenumbers at 1738 cm^−1^ and 1369 cm^−1^ correspond to the acetyl group in hemicellulose and its methyl group deformation, respectively [38,39,40]. Therefore, these results show that the *PtoCWIN4* mutant contains higher levels of cellulose and hemicellulose in the composition of secondary cell walls within xylem tissues (Figure 6B–E). To evaluate whether *PtoCWIN4* influences cellulose deposition in the secondary cell walls of the xylem, we performed calcofluor white staining to visualize mixed-linkage glucans. Comparative analysis revealed that the fluorescence intensity of cellulose in the *PtoCWIN4* mutant stem was markedly stronger than in WT plants (Figure 5I–L,N). Consistent with these findings, quantitative assessments of cell wall components demonstrated significantly elevated levels of both cellulose and hemicelluloses in *PtoCWIN4* mutants by 24.69–62.07% and 7.61–13.22% compared to the WT (Figure 6F,G). Additionally, genes involved in cellulose and the master switch in the regulation of SCW synthesis were significantly upregulated in the *PtoCWIN4* mutants (Figure 6H–P).

### 2.6. PtoCWIN4 Effectively Hydrolyzes Sucrose In Vitro

**Figure 7:**
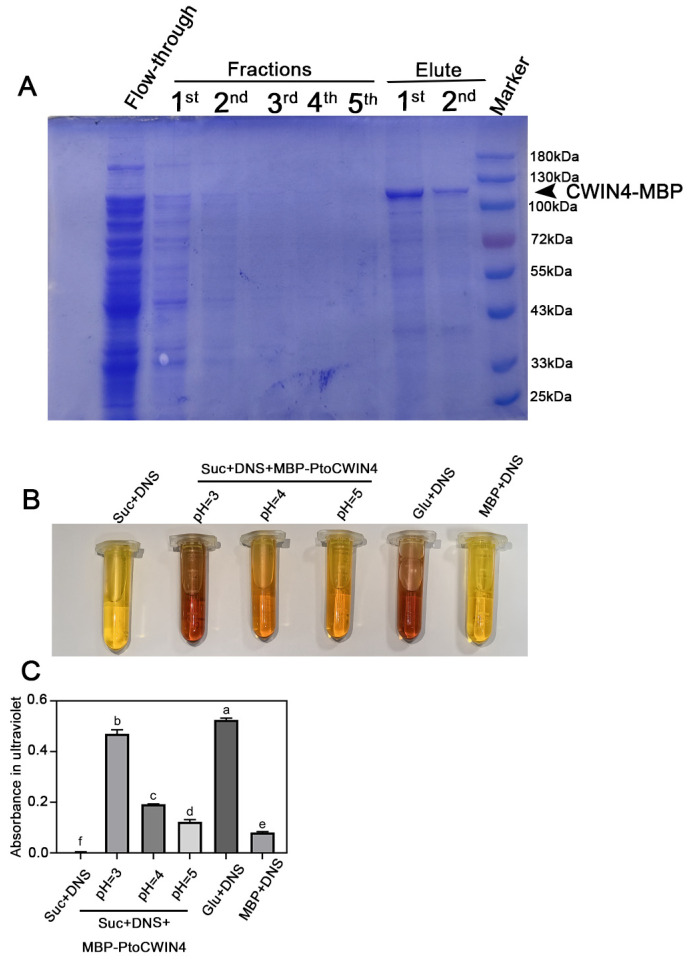
In vitro enzyme activity assay of PtoCWIN4. (**A**) Recombinant MBP-CWIN4 protein was analyzed by SDS-PAGE and Coomassie Brilliant Blue staining. (**B**) The enzymatic activity efficiency of MBP-CWIN4 in hydrolyzing sucrose was determined using the Ghose method. (**C**) The absorbency in ultraviolet quantification of DNS reaction. Different letters denote statistical differences (*p* < 0.05) as determined by one-way ANOVA. *n* = 3 for technical replicates.

To validate the sucrose-hydrolyzing capacity of PtoCWIN4, we expressed and purified the recombinant MBP-PtoCWIN4 fusion protein in a prokaryotic system (Figure 7A). SDS-PAGE analysis followed by Coomassie Brilliant Blue staining revealed a distinct protein band at ~111 kDa, consistent with the predicted molecular weight of the MBP-PtoCWIN4 fusion protein.

The enzymatic activity of PtoCWIN4 was further evaluated using the Ghose method (Figure 7B,C). The results show that MBP-PtoCWIN4 efficiently hydrolyzed sucrose, generating reducing sugars at levels comparable to those produced by direct glucose addition—as demonstrated by a proportional reddish-brown colorimetric signal—and the enzyme exhibits maximum catalytic efficiency at pH 3, with enzymatic activity sharply diminishing as the pH shifts toward 4 and 5 (Figure 7B,C). In contrast, the MBP tag alone (negative control) exhibited no detectable color change. These data conclusively demonstrate that the purified MBP-PtoCWIN4 fusion protein possesses robust sucrose-hydrolyzing activity in vitro.

### 2.7. PtoCWIN4 Affects Soluble Sugar Accumulation in Poplar Stems

**Figure 8:**
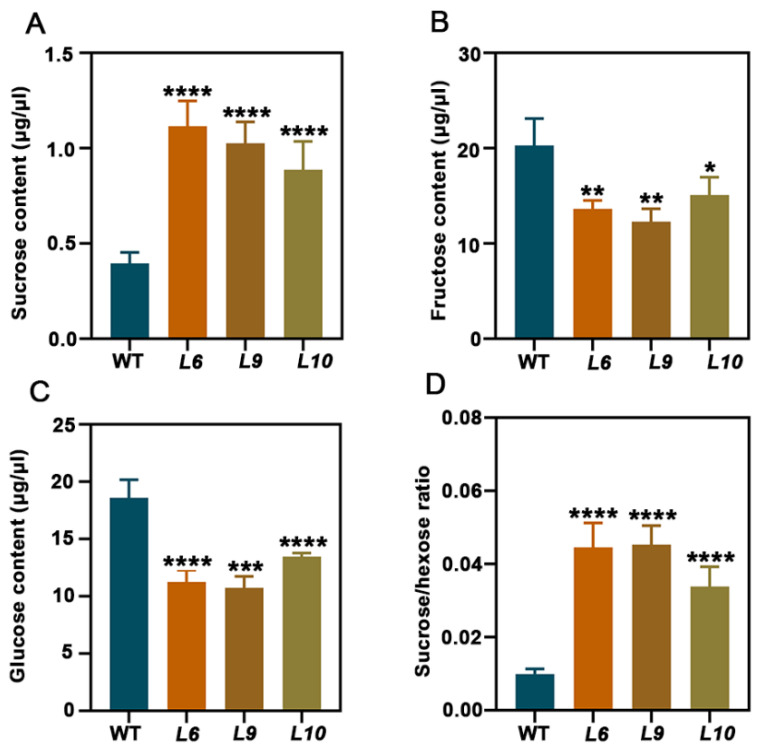
The impact of PtoCWIN4 on soluble sugar accumulation in poplar stems. (**A**–**C**) Quantification of sucrose (**A**), fructose (**B**), and glucose (**C**) content in developing wood of WT and *PtoCWIN4* mutant lines. Data are expressed as a percentage of dry weight (DW). (**D**) The ratio of sucrose-to-hexose content in WT and *PtoCWIN4* mutant lines. All data were obtained from three independent biological replicates. Error bars indicate standard deviation (**SD**). Statistical significance was determined using Student’s *t*-test (* *p* < 0.05; ** *p* < 0.01; *** *p* < 0.001, **** *p* < 0.0001; *n* = 3).

To investigate the impact of *PtoCWIN4* on sugar dynamics during wood formation, we quantified the levels of soluble sugars (sucrose, glucose, and fructose) in developing wood (Figure 8A–D). Our results show a marked alteration in carbohydrate metabolism, with sucrose concentrations increasing significantly by 121.5–178.8% in *PtoCWIN4* mutant stems compared to WT (Figure 8A). Conversely, hexose pools exhibited substantial reductions, with glucose levels decreasing by 27.6–42.5% and fructose concentrations declining by 25.5–39.4% in *PtoCWIN4* mutant plants (Figure 8B,C). The sucrose-to-hexose ratio shows a pronounced elevation (Figure 8D), demonstrating impaired carbon partitioning from sucrose to hexose metabolites during wood development. These findings collectively indicate that *PtoCWIN4* knockout perturbs sucrose metabolism toward hexose in developing wood. These metabolic shifts collectively demonstrate that *PtoCWIN4* functions as a critical regulator of sucrose hydrolysis, facilitating the conversion of sucrose to hexose intermediates essential for normal wood development.

## 3. Discussion

### 3.1. Knockout of PtoCWIN4 Increases SCW Thickness

Sugars are crucial nutrient molecules that not only provide substrates and energy for plant growth and development but also act as signaling molecules regulating these processes [3]. Consequently, abnormalities in sugar metabolism, transport, or signaling often lead to multiple defects in plant growth and development [4]. However, despite the central role of sugars in plant physiology, their specific regulatory mechanisms in wood development remain poorly understood. As a critical carbon storage organ, wood development relies on sucrose transported from source organs (e.g., leaves) through long-distance transport. The unloading and delivery of sucrose from the phloem to developing wood involves three steps: (1) symplastic unloading from the phloem into rays; (2) symplastic lateral transport within rays; and (3) export from rays to developing xylem cells [41]. Following long-distance transport from source organs, the phloem unloading and post-phloem lateral transport of sucrose in rays are essential for xylogenesis, including carbon-demanding processes such as cambium cell division and secondary cell wall (SCW) synthesis [42]. Sucrose is initially unloaded symplastically from the phloem into phloem ray cells [43]. However, sucrose unloading from rays likely occurs via an apoplastic mechanism, hypothesized to involve SWEETs mediating sucrose efflux from ray cells into the apoplastic space. Previous studies have demonstrated that the sucrose/proton symporter sucrose transporter 3 (SUT3) is expressed during secondary cell wall formation in developing wood [9,11]. In hybrid aspen (*P. tremula* × *tremuloides*), RNA interference-mediated silencing of *SUT3* in SCW-forming zones of developing wood reduced carbon incorporation into cell walls. This finding indicates that SUT3 facilitates active sucrose uptake from the apoplast [9,44], further supporting the apoplastic sucrose transport mechanism between rays and neighboring fiber cells.

Our study revealed that *PtoCWIN4* is specifically highly expressed in the apoplastic space of xylem rays, corroborating the hypothesis of sucrose efflux from xylem rays into the apoplastic space of wood. Additionally, we found that knocking out *PtoCWIN4* enhanced secondary wall synthesis. This suggests that the loss of *PtoCWIN4* reduces the conversion of apoplastic sucrose into hexoses, potentially leaving more sucrose available in the xylem apoplastic space. Subsequently, this sucrose may be imported via SUT3-mediated uptake into fibers undergoing SCW formation, thereby explaining the thickened secondary walls in *PtoCWIN4* mutants.

### 3.2. PtoCWIN4 Orchestrates Xylem Development and Branching Patterns in Poplar Through Sugar-Signaling-Mediated Metabolic Regulation

Beyond its role in sucrose unloading, CWIN mediates sugar signaling [1]. Multiple studies have revealed its regulatory functions in developmental signaling. For instance, CWIN suppression in carrot promotes leaf proliferation, while its inhibition in tomato increases petal and sepal numbers, indicating its crucial signaling role in development independent of carbon supply [45]. Notably, exogenous glucose or fructose supplementation fails to rescue developmental defects in CWIN mutants, demonstrating that these phenotypes result from impaired metabolic signaling rather than carbohydrate deficiency. This is exemplified by the irreparable miniature kernel phenotype in maize mutants [46] and the persistent pollen sterility in tobacco CWIN antisense lines [27]. Similarly, amiRNA-mediated silencing of CWIN2/4 in ovules causes abortion without inducing carbon-starvation genes, and carbohydrate supplementation cannot reverse this phenotype, confirming CWIN’s signaling function in ovule development.

CWIN also exhibits signaling roles in stress responses. Elevated CWIN activity in tomato induces pathogenesis-related (PR) gene expression and R-gene-mediated disease resistance in young fruits [47], while suppressing heat-stress-induced programmed cell death [33]. Our observations in *P. tomentosa* reveal that *PtoCWIN4* knockout severely reduces branching, consistent with Arabidopsis studies where SAM-specific CWIN overexpression accelerates flowering and enhances inflorescence branching [2]. This demonstrates *PtoCWIN4*’ s dual functionality in both sink strength regulation and developmental signaling. Mechanistically, CWIN-mediated glucose signaling activates cell-cycle-related genes [45] and auxin biosynthesis genes [48]. Our findings that *PtoCWIN4* knockout significantly reduces xylem layers suggest its involvement in regulating cambial activity. Given the tight regulation of cambium activity by multiple signaling pathways, we propose that *PtoCWIN4* likely modulates glucose signaling to coordinate cell division and auxin synthesis genes, thereby influencing cambium-derived secondary growth.

## 4. Materials and Methods

### 4.1. Plant Materials and Growth Conditions

*P. tomentosa* Carr. (Clone 741) was used in this study. The poplars were cultivated in the greenhouse under a long-day photoperiod (16 h/8 h, light/dark, 10,000 lux supplemental light), at 25 °C.

### 4.2. RNA Extraction and Real-Time Quantitative PCR

Total RNA of *P. tomentosa* plants was extracted using a Biospin Plant Total RNA Extraction Kit (Bioflux, Hangzhou, China). Complementary DNA (cDNA) was synthesized using a PrimeScript^TM^ RT reagent kit with gDNA Eraser (TaKaRa, Dalian, China). Real-time quantitative assays (RT-qPCRs) were performed by using SYBR Premix ExTaq^TM^ (TaKaRa) in a qTOWER3G IVD Real-Time PCR machine (Analytik Jena AG, Berlin, Germany). The poplar *UBIQUITIN* gene (*PtoUBQ*) was used as the reference gene. The RT-qPCR expression data were calculated using the ΔΔC_t_ method, with three individual biological replicates and three technical replicates for each gene. The primers used for the RT-qPCRs are listed in Supplemental Table S1.

### 4.3. Vector Construction and Transformation of Poplar

To generate *PtoCWIN4*-Cas9, 20-base pair designed fragments of the *PtoCWIN4* CDS were constructed to the binary pYLCRISPR/Cas9 vector as described previously [49]. To construct the *ProPtoCWIN4::GUS* vector, the promoter sequences of *CWIN4* (2524 bp upstream of ATG) were amplified from genomic DNA of *P. tomentosa* with the specific primer pairs and assembled into the pCXGUS-P vector [50].

The constructs above were stably transformed into *P. tomentosa* through *Agrobacterium*-mediated infiltration of leaf disks, as previously described [51]. The primers used in vector construction are listed in Supplemental Table S1.

CRISPR/Cas9-mediated mutation of *PtoCWIN4* in poplar was performed using the pYLCRISPR/Cas9 multiplex genome targeting system [52]. The coding region of *PtoCWIN4* was analyzed via Cas-Designer (http://www.rgenome.net/cas-designer/ (accessed on 1 February 2022)) [53,54]. Four 20-base pair sgRNA target sites (each containing a protospacer adjacent motif, PAM: 5′-NGG-3′) were designed in the first two exons of *PtoCWIN4*, with GC content ranging from 40% to 60%. These sgRNA cassettes were driven by *Arabidopsis* promoters *AtU3b*, *AtU3d*, *AtU6-1*, and *AtU6-29*, respectively. The final knockout construct (*PtoCWIN4*-KO) was assembled using Golden Gate cloning [49].

To validate the CRISPR/Cas9-induced mutations in transgenic poplar, genomic DNA was extracted using the CTAB method. The *PtoCWIN4* genomic fragment spanning the target regions was amplified with gene-specific primers (Supplementary Table S1) and cloned into the pMD19-T Simple vector (Takara, Dalian, China) for Sanger sequencing. Three transgenic lines (L6, L9, and L10) were selected for propagation. PCR genotyping and sequencing of six regenerated plants per line confirmed mutations, with more than twenty clones per line randomly sequenced.

Strict adherence to PAM (5′-NGG-3′) and GC content (40–65%) minimized off-target potential, and independent transgenic lines (L6/L9/L10) show a consistent phenotype, further supporting the on-target editing.

### 4.4. Histochemical Staining and Wood Anatomy

The 7th internodes of 2-month-old poplars were sectioned into 70 µm using a vibrating blade microtome (VT1000 s; Leica, Wezlar, Germany). Three individual poplars from WT, mutant, and transgenic lines were prepared for sectioning. Ten sections were obtained from each plant, and thirty sections in total for each line. Six sections were randomly selected for toluidine blue staining and the other six sections were used for phloroglucinol staining. For toluidine blue staining, cross-sections were stained with 0.05% (*w*/*v*) toluidine blue for 5 min. For phloroglucinol staining, cross-sections were treated with 40% H_2_SO_4_ for 30 s before staining with 1.0% (*w*/*v*) phloroglucinol for 30 s. Sections were observed and captured by a microscope camera (Olympus DP73). For phenotypic analysis, six photographs randomly generated from the six cross-sections were used for measuring the area of the xylem, the area of the stem, and the stem diameter. To determine the cell layers of cambium and xylem, 100 columns from each section were randomly selected for counting. ImageJ was used for the measurements and statistical analysis.

### 4.5. GUS Analysis

For GUS staining, the 7th internode from three individual 2-month-old GUS reporter lines were cross-sectioned and stained as previously described [55]. GUS staining was conducted using a β-glucuronidase Reporter Gene Staining Kit (Scientific Phygene, Fuzhou, China), following the manufacturer’s instructions. Stained sections were photographed by a microscope camera (Olympus DP73, Tokyo, Japan).

### 4.6. Scanning Electron Microscopy

Scanning electron microscopy analyses were conducted as previously described [55]. Cross-sections were obtained by dissecting transversely with a razor blade by hand and the samples were attached using double-sided sticky tape. The samples were observed by SEM (Phenom^tm^Pure, Massachusetts, USA) following the manual’s recommendations, and images were captured digitally at 15 KV voltage.

### 4.7. Calcofluor White Staining

Calcofluor white staining was conducted as previously described [56]. The 7th internode from three individual 2-month-old WT, mutant, and transgenic lines were cross-sectioned and stained with 0.1% (*w*/*v*) calcofluor white for 5 min and washed with 0.5 M mannitol (pH 7.0) 3 times. Afterward, they were mounted with 0.5 M mannitol for imaging under a confocal microscope.

### 4.8. Confocal Microscopy

This includes the calcofluor white fluorescence analysis, as previously described [56].

Samples were imaged by a confocal laser scanning microscope (FV3000 Olympus, Tokyo, Japan) equipped with the following filter sets: 350/450 nm (ex/em) for visualizing calcofluor-white-stained cell walls.

### 4.9. SCW Composition Determination

The total stems of the 2-month-old poplars were harvested and dried at 50 °C. Dried samples were ground into a powder that can pass through a 40-mesh sieve, keeping it dry until use. Cellulose and hemicellulose quantification assays were conducted, as previously described [56].

FTIR spectroscopy was conducted to analyze the chemical linkage in SCW, as described by [55]. The stem (after removing the epidermis, phloem, and pith) of 2-month-old poplars were dried and ground into a powder that can pass through a 60-mesh sieve. KBr was added to samples at a 100:1 ratio and heated at 103 °C for 24 h. The mixture was ground in an agate mortar under infrared lamplight and compressed into tablets (13 mm in diameter). Samples were determined by Fourier-transform infrared spectroscopy (INVENIO-S of Bruker, Ettlingen, Germany). Spectral acquisitions were performed in transmission mode. Spectrum range: 4000 ~ 400 cm^−1^; resolution: 16 cm^−1^; 32 acquisition points sweeping each tablet. Three individuals were analyzed for each genotype—ten technical replicates for each individual. After sorting the spectra and correcting the baseline, the spectra were area-normalized and the different genotypes were compared for the absorbance values of major cellulose and hemicellulose bands in the fingerprint region (1800–800^−1^). OPUS and Origin were used for the data collection and processing.

### 4.10. Prokaryotic Expression and Purification of MBP-PtoCWIN4 Protein

The construct containing the MBP-PtoCWIN4 was transformed into the *E. coli* prokaryotic protein expression strain BL21(DE3) using the heat-shock method. The positive monoclonal colonies were selected and PCR-confirmed. The positive single colony was transferred into 5 mL of LB liquid medium containing 100 μg/mL ampicillin. The culture was incubated at 37 °C, shaking at 220 rpm for 12–16 h. The next day, the culture was inoculated at a 1:50 ratio into 1 L of LB liquid medium containing 100 μg/mL ampicillin. The cells were grown at 37 °C, shaking at 200 rpm until the OD600 reached approximately 0.8. Protein expression was induced by adding 0.5 mM IPTG, followed by continued incubation at 16 °C, shaking at 180 rpm for 12–16 h to express the MBP-PtoCWIN4 protein.

Cells were harvested by centrifugation at 4000 rpm for 10 min at 4 °C. The pellet was resuspended in 15 mL of 1× PBS buffer (0.2 g KCl, 8 g NaCl, 1.42 g Na_2_HPO_4_, and 0.27 g KH_2_PO_4_ per 1 L) and stored in a 50 mL centrifuge tube. Then, the resuspended cells were lysed using an ice-bath-assisted sonicator (SCIENTZ-IID, Ningbo, China). Sonication was performed at 200 W with cycles of 3 s on and 7 s off for a total duration of 45 min until the lysate became clear. This was centrifuged at 4 °C and 5000 rpm for 25 min, then the supernatant (cell lysate) was collected to remove the cell debris. An appropriate amount of MBPSep Dextrin Agarose Resin 6FF was loaded into a chromatography column. The resin was equilibrated with 5 column volumes of equilibration buffer (20 mM Tris-HCl, 200 mM NaCl, 1 mM EDTA, pH 7.4) to match the buffer system of the target protein, protecting protein stability. The lysate was then applied to the column and incubated at 4 °C with rotation for 1–2 h, after which the flow-through was collected. The column was washed with 10–15 column volumes of wash buffer (20 mM Tris-HCl, 200 mM NaCl, 1 mM EDTA, pH 7.4), and the wash fractions were collected. This washing step was repeated 5 times. Then, the column was washed with 10–15 column volumes of elution buffer (20 mM Tris-HCl, 1 mM EDTA, 10 mM maltose, pH 7.4) and the wash elution was collected. This washing step was repeated 2 times. The eluted target protein was analyzed by SDS-PAGE, and its concentration was determined using the Bradford assay.

The purified MBP-PtoCWIN4 protein was used for DNS reagent-based detection of its sucrose hydrolase activity (TC0028, Beijing Leagene Biotechnology, Beijing, China). The MBP-PtoCWIN4 protein was loaded into a dialysis bag (FDM303-5 m, Beyotime, Shanghai, China) and dialyzed against a buffer (1.25 M EPES, 0.5 M EDTA, pH 8.0) with three buffer changes (4–5 h each) to remove residual maltose. For the reaction, 20 ng of MBP-PtoCWIN4 protein was mixed with 3 mg of sucrose and 1 mL of DNS reagent, and the pH was adjusted to 3, 4, and 5, respectively. The controls included the following: 3 mg sucrose + 1 mL DNS reagent; 3 mg glucose + 1 mL DNS reagent; 20 ng MBP protein + DNS reagent. The reactions were incubated at 37 °C for 40 min, followed by boiling for 5 min. Absorbance was measured by microplate reader at 540 nm (SpectraMax^®^ 190, Molecular Devices, San Jose, CA, USA).

### 4.11. Soluble Sugar Analysis

To determine the soluble sugars, the stem of 2-month-old poplars of WT, transgenic, and mutant lines were collected. The soluble sugars were extracted as described by Willige et al. (2009) [57]. Plant material was ground to a fine powder using liquid nitrogen, freeze-dried, and then extracted twice with 80% (*v*/*v*) methanol (3 mL g^−1^) at 4 °C. The homogenates were cleared by centrifugation (5000× *g*, 5 min, 4 °C). At 25 °C, the methanol evaporated until it was completely dry under low pressure. The sediment was taken up in water and washed three times with chloroform to remove lipophilic substances. To remove any particles, the aqueous phase was centrifuged for 30 min at 10,000× *g* and 4 °C. A cation (Dowex 50 WX8) and an anion (Dowex IX8) exchange resin were added to the aqueous phase (5 g resin per 100 mL) to remove organic acids, amino acids, or other charged molecules. After stirring for 1 h, the aqueous phase was transferred into a reaction vial for GC analysis. The extracted sugar fractions were further separated and identified by coupled gas chromatography/mass spectrometry (GC/MS). A total of 10 µL of extract, prepared as described above, and 1.5 µg of xylitol (used as the internal standard) were dried at 60 °C under N_2_ gas. Amounts of 30 µL of pyridine and 30 µL of N, O-Bis (trimethylsilyl) trifluoracetamide (BSTFA) were then added and the sample was diluted with 50 µL of chloroform. The samples were heated at 70 °C for 40 min. The trimethyl silyl (TMS) sugar derivatives were separated on a TG-SQC column (Thermo Scientific, Folsom, CA, USA). Qualitative GC/MS analysis was carried out with a gas chromatograph TSQ9610, detector TRACE1600 (Thermo Scientific). In total, 1 µL of each sample was injected and the flow was set to 5 mL/min. The initial temperature was 65 °C for 3 min, after which the temperature was raised at a rate of 8 °C min^−1^ to a temperature of 240 °C, after which the rate was increased by 12 °C/min to a final temperature of 310 °C for 35 min. Data analysis was performed with Thermo Fisher Scientific-CN–Chromeleon. According to the standard curve of glucose, fructose, and sucrose, the content of each sugar in the sample was calculated by the peak area. The representative spectral peaks for soluble sugar quantification are shown in Supplemental Figure S1.

### 4.12. Statistical Analyses

The data presented in this study were examined for statistically significant differences using Student’s *t*-test (GraphPad Prism software 7.04; GraphPad Software, Boston, MA, USA) or a one-way ANOVA (IBM SPSS Statistics 22.0; IBM, Armonk, NY, USA), as described in the corresponding figure legends. Error bars indicate standard deviation. The least significance difference (LSD) test was adopted for all pairwise comparisons in the one-way ANOVA analysis, with *p* values < 0.05 considered significant differences.

## Data Availability

The original contributions presented in this study are included in the article/Supplementary Material. Further inquiries can be directed to the corresponding author(s).

## Supplementary Materials

The following supporting information can be downloaded at https://www.mdpi.com/article/10.3390/plants14091388/s1, Figure S1: The representative spectral peaks for soluble sugar quantification assay; Table S1: Primers used for experiments.
