# Supplementary material for: Cell Wall Invertase 4 Governs Sucrose–Hexose Homeostasis in the Apoplast to Regulate Wood Development in Poplar
Source: Plants (Basel). 2025 May 4;14(9):1388. doi: 10.3390/plants14091388 (PMC12073565; doi:10.3390/plants14091388)
Supplement: Supplementary file 1 [file plants-14-01388-s001.zip › Supplemental Figure S1.pdf]

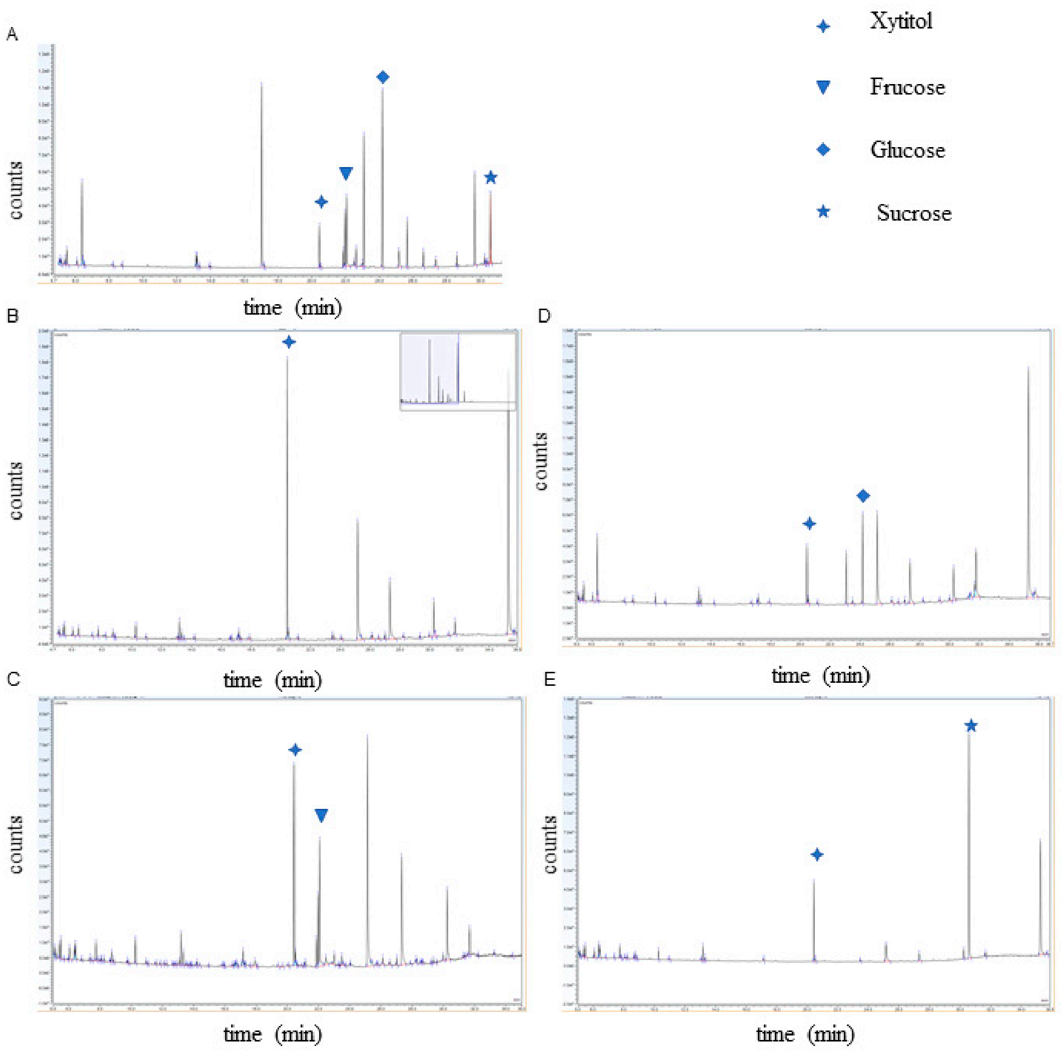

Supplemental Figure S1. The representative spectra peak for soluble sugar quantification assay by GC-MS. (A). Characteristic GC-MS spectra peak of soluble sugars in the sample; (B-E). Characteristic GC-MS spectra peak of the xytitol standard(B); fructose standard(C); glucose standard(D); sucrose standard(E).
