# Supplementary material for: Cell Wall Invertase 4 Governs Sucrose–Hexose Homeostasis in the Apoplast to Regulate Wood Development in Poplar
Source: Plants (Basel). 2025 May 4;14(9):1388. doi: 10.3390/plants14091388 (PMC12073565; doi:10.3390/plants14091388)
Supplement: Supplementary file 1 [file plants-14-01388-s001.zip › Supplemental Table S1.pdf]

Supplemental Table S1. Primers used for experiments

Name	Forward primer (5'-3')	Reverse primer (5'-3')
UBQ	GTTGATTTTTGCTGGGAAGC	GATCTTGGCCTTCACGTTGT
RT-qRCP-PtoCIN1	ATGGATGTTGGTCTGGCTCA	TACGTTAGCATCCGGGTTCA
RT-qRCP-PtoCIN2	CGCAAACTTGTCGGATCCAT	GTGTTTGGTCTTGGCCCATT
RT-qRCP-PtoCIN3	AGAACAAAGGATGGGCAGGA	AGGATCAAAGGGCTCAGCTT
RT-qRCP-PtoCIN4	CTTCGAGATGAGGTCGATAAA	TTAGGATAAACTCTATTGGTGATG
RT-qRCP-PtoCIN5	GGTGGTTGGATTCTGCGTTT	TATGACACCGAATGAGCCCA
PtoCIN4pro-GUS	CGGGGATCCTCTAGAGGTAATCTAGGTC ATGTTTGTTTCTC	CTTGCATGCCTGCAGGGTTTGCATCTA TCACAAAGACTG
cas9-PtoCIN4-AtU3b	gtcAGTTGAAGCCTCGGAGAATAACGG	aaacCCGTTATTCTCCGAGGCTTCAAC
cas9-PtoCIN4-AtU3d	gtcACCTTTCACTTCCAACCACCAAGA	aaacGCTACTGCAACAATATACTG
cas9-PtoCIN4-AtU6-1	attGAATCTAGTGCAGGGCCAATGTGG	aaacCCACATTGGCCCTGCACTAGATT
cas9-PtoCIN4-AtU6-29	attG CCTTTGCCCAACGGAACCATATG	aaacCATATGGTTCCGTTGGGCAAAGG
RT-qRCP-PtoCesA4	GTGTGTGGCTTTCCTGTTTGTAG	GGCATCCTCATCGTCATTATCTCC
RT-qRCP-PtoCesA7	GTTTTCCTGCGTGTAGACCA	TTATTCTTGTCTTGCTCATCTTC
RT-qRCP-PtoCesA8	GTTGGCCTCTGTCTTCTCTCTTGTC	CAATCTATAGAAATGCAGGTTTCAC
RT-qRCP-PtoMYB021	CGGAGCCTAGCAAAGATGTC	ATTGCTTTCATGGAGGTTGC
RT-qRCP-PtoSND1-A1	TAGGCTTGATGACAGCACCCATGAA	TCTAAATACCCGGCAAACCACCC AA
RT-qRCP-PtoSND1-A2	TCCGGGCAACTTAACGATTGGGTA	GCATTTGGGCCGGTAGTAAAGCA
RT-qRCP-PtoKANT7	TGACATCCTTGAAGTCCAAG	CTTGGACTTCAAGGATGTCA
RT-qRCP-PtoVND6A2	CGACGAAGAACTTGTCGATTAC	TCATTTTGTTCCTCGGTTCCTA
RT-qRCP-PtoWND2A	AACTGGGCAACCCTTGATCGTCTA	GTAATGGTTGGGTCAATGCAGGGT
MBP-PtoCIN4	GAGGGAAGGATTTCAGAATTCATGGAG ATTCTTGCTGTT	CAGGTCGACTCTAGAGGATCCTTAAA TGAAGTTTTCTTT
